# Selective internal radiotherapy (SIRT) versus transarterial chemoembolization (TACE) for the treatment of intrahepatic cholangiocellular carcinoma (CCC): study protocol for a randomized controlled trial

**DOI:** 10.1186/1745-6215-15-311

**Published:** 2014-08-06

**Authors:** Roman Kloeckner, Christian Ruckes, Kai Kronfeld, Marcus Alexander Wörns, Arndt Weinmann, Peter Robert Galle, Hauke Lang, Gerd Otto, Waltraud Eichhorn, Mathias Schreckenberger, Christoph Dueber, Michael Bernhard Pitton

**Affiliations:** Department of Diagnostic and Interventional Radiology, Johannes Gutenberg-University Medical Center, Langenbeckstr. 1, 55131 Mainz, Germany; Interdisciplinary Center for Clinical Trials (IZKS), Johannes Gutenberg-University Medical Center, Langenbeckstr.1, 55131 Mainz, Germany; First Department of Internal Medicine, Johannes Gutenberg-University Medical Center, Langenbeckstr.1, 55131 Mainz, Germany; Department of General and Transplant Surgery, Johannes Gutenberg-University Medical Center, Langenbeckstr.1, 55131 Mainz, Germany; Department of Nuclear Medicine, Johannes Gutenberg-University Medical Center, Langenbeckstr.1, 55131 Mainz, Germany

**Keywords:** Intrahepatic cholangiocellular carcinoma (CCC), loco-regional treatment, selective internal radiotherapy (SIRT), transarterial chemoembolization (TACE)

## Abstract

**Background:**

Cholangiocellular carcinoma is the second most common primary liver cancer after hepatocellular carcinoma. Over the last 30 years, the incidence of intrahepatic cholangiocellular carcinoma has risen continuously worldwide. Meanwhile, the intrahepatic cholangiocellular carcinoma has become more common than the extrahepatic growth type and currently accounts for 10-15% of all primary hepatic malignancies. Intrahepatic cholangiocellular carcinoma is typically diagnosed in advanced stages due to late clinical symptoms and an absence of classic risk factors. A late diagnosis precludes curative surgical resection. There is evidence that transarterial chemoembolization leads to better local tumor control and prolongs survival compared to systemic chemotherapy. New data indicates that selective internal radiotherapy, also referred to as radioembolization, provides promising results for treating intrahepatic cholangiocellular carcinoma.

**Methods/Design:**

This pilot study is a randomized, controlled, single center, phase II trial. Twenty-four patients with intrahepatic cholangiocellular carcinoma will be randomized in a 1:1 ratio to receive either chemoembolization or radioembolization. Randomization will be stratified according to tumor load. Progression-free survival is the primary endpoint; overall survival and time to progression are secondary endpoints. To evaluate treatment success, patients will receive contrast enhanced magnetic resonance imaging every 3 months.

**Discussion:**

Currently, chemoembolization is routinely performed in many centers instead of systemic chemotherapy for treating intrahepatic cholangiocellular carcinoma confined to the liver. Recently, radioembolization has been increasingly applied to cholangiocellular carcinoma as second line therapy after TACE failure or even as an alternative first line therapy. Nonetheless, no randomized studies have compared radioembolization and chemoembolization. Considering all this background information, we recognized a strong need for a randomized controlled trial (RCT) to compare the two treatments. Therefore, the present protocol describes the design of a RCT that compares SIRT and TACE as the first line therapy for inoperable CCC confined to the liver.

**Trial registration:**

ClinicalTrials.gov, Identifier: NCT01798147, registered 16^th^ of February 2013.

## Background

### Epidemiology

Cholangiocellular carcinoma (CCC) is the second most common primary liver cancer after hepatocellular carcinoma (HCC) [1]. CCCs are classified according to their location; they are either intrahepatic or extrahepatic hilar CCCs [2]. Over the last 30 years, the incidence of intrahepatic CCC has risen continuously worldwide [3–5]. In 1975 to 1979, the incidence was only 0.32 per 100,000 people; by 1995 to 1999, the incidence had risen to 0.85 per 100,000 people. Moreover, the intrahepatic CCC subtype became more common than the extrahepatic subtype [6]. Currently, the intrahepatic subtype accounts for 10 to 15% of primary hepatic malignancies [7].

### Diagnosis

Only a few patients present typical risk factors: the majority of cases develop spontaneously in patients with non-cirrhotic liver [5, 8–18]. There are no early changes in standard blood parameters. This relative independence from classic risk factors, and the lack of jaundice and early clinical symptoms typically delays diagnosis [2, 19]. Currently, ultrasound is the most frequently performed imaging method [20]. Nonetheless, there are only a few specific diagnostic criteria for intrahepatic CCC [21, 22], and inter-observer variability is high [23]. Therefore, cross-sectional imaging with computed tomography (CT) or magnetic resonance imaging (MRI) is mandatory.

#### Computed tomography

Contrast-enhanced CT is a widely used method for the primary diagnosis and staging of CCC [22]. CT allows evaluation of the primary tumor and can rule out extrahepatic metastasis [24]. Typical imaging features are nodular, irregularly shaped intrahepatic masses, which show moderate contrast enhancement in the arterial and portal venous phases [22].

#### Magnetic resonance imaging

Due to its high soft-tissue contrast, MRI with an intravenous contrast medium is the modality of choice for staging of intrahepatic CCC [25, 26]. In analogy to CT, the tumor shows only moderate contrast enhancement in the arterial and portal venous phases and prolonged enhancement in the late phases [27, 28].

### Prognosis

The prognosis of intrahepatic CCC is poor [5, 6, 29]. In the last few decades, a small improvement was observed in the 1-year survival rate, which rose from 16.4% (1975 to 1979) to 27.6% (1995 to 1999). This improvement was mainly due to better supportive care. The main reason for the poor prognosis is the late diagnosis leading to advanced stages [6]. Prognosis is correlated with the tumor stage and lymph node status (5-year survival rates for patients with lymphatic infiltration versus those without were 44% versus 11%, respectively) [30].

### Treatments

#### Resection

Surgical resection is the treatment of choice [5]. When a complete removal (R0-resection) is accomplished, 3-year survival rates of 40 to 60% are reported [31–38]. However, when the tumor is incompletely removed, the results are not better than those obtained with palliative care [39].

#### Local ablative therapy

Patients with very few but clearly definable lesions can be treated with local ablative therapies like radiofrequency or microwave ablation. The intention is to completely ablate the tumor, including a safety margin of a least 1 cm [40]. Because 5 cm of liver tissue is the maximum diameter that can be safely ablated with the standard probes available, 3 cm is typically the maximum tumor diameter suggested for ablation.

#### Chemotherapy

Survival was proven to be higher with Cisplatin and Gemcitabine compared to Gemcitabine monotherapy [41]. Nonetheless, only 59% of patients in that study had extra- or intrahepatic CCC; the remainder had tumors in papillary tissues and the gallbladder. Therefore, those results were only partially applicable to patients with intrahepatic CCC.

#### Transarterial chemoembolization

Transarterial chemoembolization (TACE) is based on delivering small particles into the tumor feeding arteries via a catheter to cause embolization or occlusion of the tumor’s arterial supply. The particles also contain a chemotherapeutic agent for specific tumor treatment. In inoperable patients, survival was greater with TACE than with best supportive care [42–44]. Recently published data indicate that TACE performed with drug-eluting beads (DEB TACE) lead to better local tumor control and both prolonged progression-free survival (PFS) and overall survival (OS) compared to systemic chemotherapy [45]. The rate of side effects was acceptable; thus, TACE was considered a good method for preserving quality of life for as long as possible [42, 45, 46].

### The need for a trial

As indicated above, the only curative therapies for intrahepatic CCC are surgical resection with tumor-free margins [39] or local ablative therapy with a sufficient safety margin [40]. Unfortunately, most patients are initially diagnosed in an advanced stage, which prohibits a curative approach. In the palliative setting, no treatment alternatives can provide satisfactory results. In the advanced stages, with extrahepatic spread of the tumor, systemic chemotherapy is the treatment of choice. When the tumor is still confined to the liver, TACE can serve as an alternative to systemic chemotherapy, with lower side effects and a considerably higher local antitumor effect [44, 45, 47].

Over the last few years, selective internal radiotherapy (SIRT) has been increasingly used as a second-line therapy for HCC treatment in case of TACE failure. Basically, SIRT is based on the same principles as TACE. The main difference is that SIRT relies on smaller-sized particles that are loaded with a radioactive beta emitter leading to an internal radiation therapy with very high local doses. One of its main advantages is the reduced number of treatments needed compared to TACE (1 to 2 versus 4 to 10 treatments) and the enhanced quality of life [48]. Additionally, repetitive TACE may be associated with considerable vessel damage. Therefore, SIRT has been increasingly discussed as a possible first-line therapy option for patients with CCC confined to the liver. New data has already shown promising results for patients with intrahepatic CCC treated with SIRT [49]. The reduced number of treatment sessions and the smaller size of the particles preserve the patency of the tumor feeding arteries. Consequently, direct access to tumor vessels is maintained, which enables the application of another local treatment (for example, TACE as a second-line treatment) in case of SIRT failure.

No randomized study has been published on the treatment of CCC with SIRT. Considering all this background information, we recognized a strong need for a randomized controlled trial (RCT) to compare the two treatments. Therefore, the present protocol describes the design of an RCT that compares SIRT and TACE as the first-line therapy for inoperable CCC confined to the liver.

## Methods/Design

### Trial center and study registration

This is a prospective, single-center, randomized pilot study with two parallel treatment groups that receive either DEB TACE or SIRT. Stratification will be carried out according to tumor load (<25% and ≥25%). The study will be performed according to the principles stated in the revised version of the Declaration of Helsinki. The study has been approved by the responsible ethics committee (Ethics Committee of the Medical Chamber of the Federal State of Rhineland Palatinate, Germany). The reference number is 837.527.10 (7536).

### Participants

The target study population is 24 patients, 12 patients in each group. If patient numbers in both groups are unequal, for example, due to dropouts, recruitment will be continued until the smaller group includes 12 patients. Written informed consent will be obtained from each patient.

### Inclusion/exclusion criteria

Inclusion criteria are: age ≥18 years; intrahepatic CCC, proven by histology or by typical morphology in cross sectional imaging and elevated tumor markers (CEA or CA 19–9); tumor confined to the liver; at least one measurable lesion detected on MRI; tumor load ≤50%; preserved liver function (Child Pugh A and B).

Exclusion criteria are: patients eligible for curative treatment (resection or local ablation); previous TACE or SIRT; prior chemotherapy; Child Pugh stage C; Eastern Cooperative Oncology Group (ECOG) performance status >1; Tumor involvement >50% of the liver; extrahepatic tumor spread; serum bilirubin >2.0 mg/dl; serum albumin >2.8 g/dl; serum creatinine >2 mg/dl; leukocytes <3,000/ml; thrombocytes <50,000/ml; clinically apparent ascites (ascites only detectable on the CT/MRI is not considered an exclusion criterion); esophageal bleeding during the last 3 months; hepatic encephalopathy; transjugular intrahepatic portosystemic shunt; any infiltration or occlusion of the portal vein (including the left, right, and main branches); hepatofugal blood flow in the portal vein; hepatopulmonary shunt ≥20% in the macroaggregated albumin (MAA) scan; contraindications against angiography; and pregnancy.

### Trial design

#### Experimental intervention

SIRT is a locoregional treatment that selectively irradiates liver tumors. The particles are loaded with the beta emitter Yttrium 90 (Y-90) and are delivered to the hepatic artery or its (sub)-segmental branches in order to embolize and selectively irradiate the tumor. Due to the short range of beta radiation, the tumor is predominantly targeted, and most of the normal liver tissue is spared. The procedure is performed once in each liver lobe. SIRT particles are commercially provided by two companies, and both are acceptable for a prospective study protocol. Based on our significant experience with one of these products, we will use SirSpheres® in this study. The most serious complication associated with SIRT is radiation-induced liver disease, which is, in fact, radiation-induced liver failure; this condition leads to death within a few weeks [50, 51]. To minimize this complication, the right and left liver lobes will be treated sequentially, with a between-treatment interval of 4 weeks. The liver lobe with the larger tumor mass will be treated first. The dose for each liver lobe will be calculated according to the body surface method [51, 52]. This is the most frequently used approach for resin spheres (73.5% in a large series of 680 treatments) [53], and it is recommended by the manufacturer, SIRTEX, and by the Radioembolization Brachytherapy Oncology Consortium (REBOC) [52].

Patients randomized to SIRT will receive a preparative intervention, including embolization of the collateral arteries (mainly the gastroduodenal artery and the right gastric artery), and subsequently, an MAA scan will be performed. Follow-up visits will be performed at 4 and 12 weeks after completion of SIRT treatment, and every 3 months thereafter until the clinical endpoints are reached. In cases where local tumor progression is detected and no contraindications exist, SIRT can be repeated once in each lobe. In cases where contraindications exist, the patient is allowed to cross over to TACE if there are no contraindications against TACE.

#### Control intervention

TACE is a well-established alternative to classic chemotherapy in patients with tumors confined to the liver; in this trial, TACE will serve as the control arm. Numerous different TACE techniques have been reported in the literature for treating HCC and CCC [44, 47, 54]. Since 2006, doxorubicin-loaded DEBs have been commercially available in various sizes. Compared to conventional TACE, which uses Lipiodol mixed with a chemotherapeutic agent, DEBs have been shown to be more effective, with fewer side effects in patients with HCC [55, 56]. Currently, DEB TACE is the only TACE method that is standardized in terms of the embolization material, the chemotherapeutic agent, and the application technique [55, 57]. Because we have considerable experience with DcBeads®, we will continue to use them exclusively throughout the trial to ensure reproducibility. The bead size will be 100 to 300 μm.

An optimal dose of 150 mg doxorubicin per application was determined in a prospective trial in patients with HCC [58]; there will be no dose adjustment made for elevated bilirubin or body surface area. After mixing with nonionic contrast medium, the DcBeads are to be administered slowly, and as selectively as possible with a microcatheter under fluoroscopic control. The mixture will be infused until the flow is sluggish to avoid reflux. In cases of incomplete embolization, additional bland (drug-free) embolization is not permitted. The TACE procedure will be repeated every 6 weeks as this interval has been established in several clinical trials for HCC and intrahepatic CCC [44, 47, 55, 56, 59]. TACE treatment will be carried out until either no viable tumor tissue is detected by MRI or if any contraindications against repeated treatment occur (for example relevant ascites, portal invasion or occlusion of the arterial feeding vessels). Therefore, additional MRI is indicated prior to each TACE treatment. After cessation of TACE treatment, follow up will be carried on in 3-month intervals until the clinical endpoints are reached. In cases where local tumor progression is detected and no contraindications exist, TACE can be repeated. In cases where contraindications exist, the patient is allowed to cross over to SIRT if there are no contraindications against SIRT.

#### Follow-up

Each follow up will include MRI of the liver, a quality-of-life assessment, documentation of adverse/serious adverse events, a physical examination, and blood tests. All MRI examinations will be performed according to a standardized protocol of MRI sequences, including transversal T1- and T2-weighted images, diffusion-weighted images, and multiphasic contrast-enhanced T1-weighted images. All examinations will be performed on a single 3 T scanner (Skyra®, Siemens Healthcare, Erlangen, Germany).

#### Outcome measures

**Figure 1:**
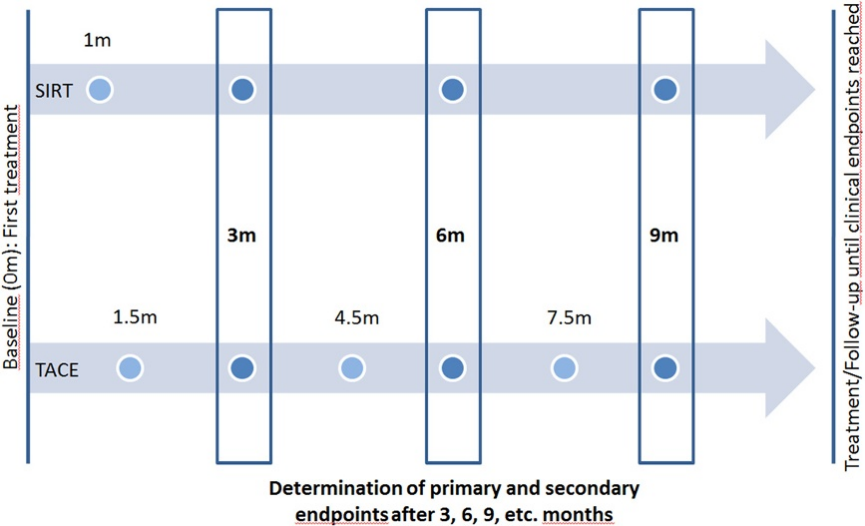
**Treatment and follow-up schedule.** Follow up begins after the completion of the first treatment. For determination of progression-free survival (PFS) only the magnetic resonance imaging (MRI) performed at 3, 6, 9 months et cetera, will be used in both groups.

PFS is directly related to the local treatment result. The local tumor response will be measured with the modified response evaluation criteria in solid tumors (mRECIST) on the basis of the MRI [60]. In an attempt to eliminate any potential bias that could derive from the difference in follow-up intervals, for determination of PFS only the MRI performed at 3, 6, 9 months et cetera, after the first treatment will be used in both groups (Figure 1). However, survival is limited by both tumor progression and liver performance. Because both treatments also affect parts of the normal liver parenchyma, they can potentially lead to liver failure. Additionally, extra-hepatic metastases develop more often in patients with intra-hepatic CCC than in patients with HCC. Therefore, PFS will be the primary endpoint, because it includes the local treatment effect, death by deteriorating liver function due to liver toxicity, and death due to extra-hepatic tumor spread.

OS is also influenced by drug-induced impaired liver function, by extra-hepatic tumor spread, and by secondary treatment strategies applied after the SIRT/TACE treatment. Therefore, OS is considered a secondary endpoint. Time to progression (TTP) serves as another secondary endpoint. The local tumor response will be determined on the basis of the MRI performed at 3, 6, 9 months et cetera, after the first treatment.

#### Determination of primary and secondary measures

As indicated above, the local tumor response will be measured according to the detailed mRECIST criteria on the basis of the MRIs performed at 3, 6, 9 months et cetera, after the first treatment [60]. Progression is defined as tumor progression that cannot be properly treated by the respective local treatment method. The final decision that progression cannot be treated properly with the respective local treatment method will be at the discretion of the investigators after consulting with an internal tumor conference and the interdisciplinary study team (including members from Surgery, Hepatology, Oncology, and Nuclear Medicine). Performance status, evaluated according to the Eastern Cooperative Oncology Group (ECOG), will be assessed during the follow-up visits by physical examination.

#### Quality assurance

To ensure quality execution, patient welfare, and compliance with ethical and legal stipulations, the Interdisciplinary Center for Clinical Trials (IZKS), Mainz, will perform the regulatory tasks (support in the development and review of study-related documents) and statistical analysis (carried out by an independent statistician). These functions will be performed according to the standard operating procedures of IZKS, which are based on the guidelines stated at the International Conference on Harmonization of Technical Requirements for the Registration of Pharmaceuticals for Human Use and good clinical practice.

#### Statistical analysis

The analysis of the study will be performed in a purely exploratory manner. Statistically significant results are unlikely unless the difference between the two methods is greater than expected. The main purpose of the analysis is to provide estimates of PFS, OS, and TTP. In particular, the time-to-event data will be analyzed with Kaplan-Meier methods. Descriptive statistics of all analyzed parameters will be provided whenever appropriate.

#### Power calculation/analysis

In the absence of previous randomized trials or data regarding SIRT as a first-line therapy for patients with intrahepatic CCC, it is not possible to perform a reliable power calculation. Therefore, this trial is designed as a pilot study.

## Discussion

To the best of our knowledge this will be the first randomized trial to compare SIRT and TACE for the treatment of intra-hepatic CCC. A finding that there are significant differences between SIRT and TACE will provide a means to improve clinical decisions regarding treatment, and it will directly influence future treatment costs for health insurance companies. The superiority or even non-inferiority of SIRT would directly impact patient comfort due to the reduced number of treatment sessions and the preservation of tumor vessel patency for secondary TACE in cases of potential SIRT failure. If this trial reveals no significant findings, the results can be used to properly calculate the number of patients required for a future confirmatory trial.

## Trial status

This trial is currently open for recruitment. To date, 12 patients have been included in the study; 6 were randomized to the SIRT group and 6 to the TACE group. One patient in the SIRT group dropped out before treatment because he developed multiple cholangitic abscesses. To date, there have been no dropouts in the TACE group. Because intra-hepatic CCC is a relatively rare tumor, recruitment is not expected to be completed before Q3 of 2016.

## Abbreviations

CCC: cholangiocellular carcinoma
CT: computed tomography
DEB: drug-eluting beads
HCC: hepatocellular carcinoma
IZKS: Interdisciplinary Center for Clinical Trials, Mainz
MAA-scan: macroaggregated albumin scan
mRECIST: modified response evaluation criteria in solid tumors
MRI: magnetic resonance imaging
OS: overall survival
PFS: progression-free survival
RCT: randomized controlled trial
SIRT: selective internal radiotherapy
TACE: transarterial chemoembolization
Y-90: Yttrium 90.
